# Supplementary material for: Immunization of mice with chimeric antigens displaying selected epitopes confers protection against intestinal colonization and renal damage caused by Shiga toxin-producing Escherichia coli
Source: NPJ Vaccines. 2020 Mar 12;5:20. doi: 10.1038/s41541-020-0168-7 (PMC7067774; doi:10.1038/s41541-020-0168-7)
Supplement: Supplementary file 1 — Supplementary Data [file 41541_2020_168_MOESM1_ESM.pdf]

SDS-PAGE & Western blot Analysis: Chimera 1

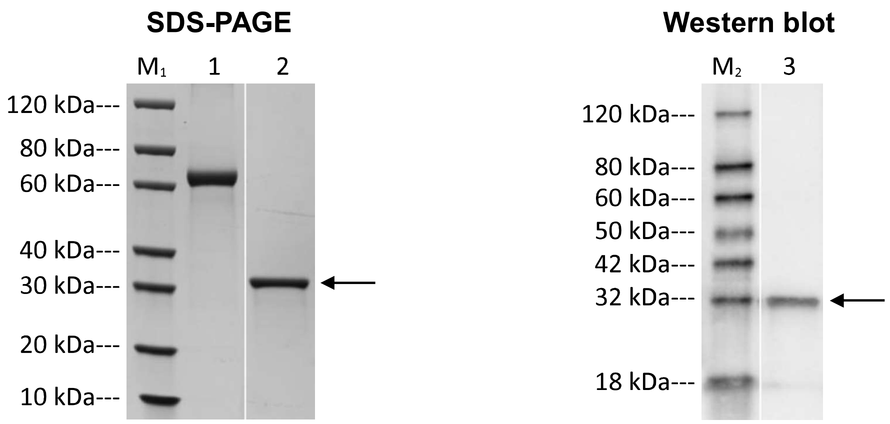

Fig.1 SDS-PAGE and Western blot analysis of PROT4 O_H
Lane M_1: Protein Marker, GenScript, Cat. No. M00516
Lane M_2: Protein Marker, GenScript, Cat. No. M00521
Lane 1: BSA (2.00 µg)
Lane 2: PROT4 O_H (Reducing condition, 2.00 µg)
Lane 3: PROT4 O_H (Reducing condition)
Primary antibody: Mouse-anti-His mAb (GenScript, Cat.No. A00186)

Quantification--BSA Standard Curve:

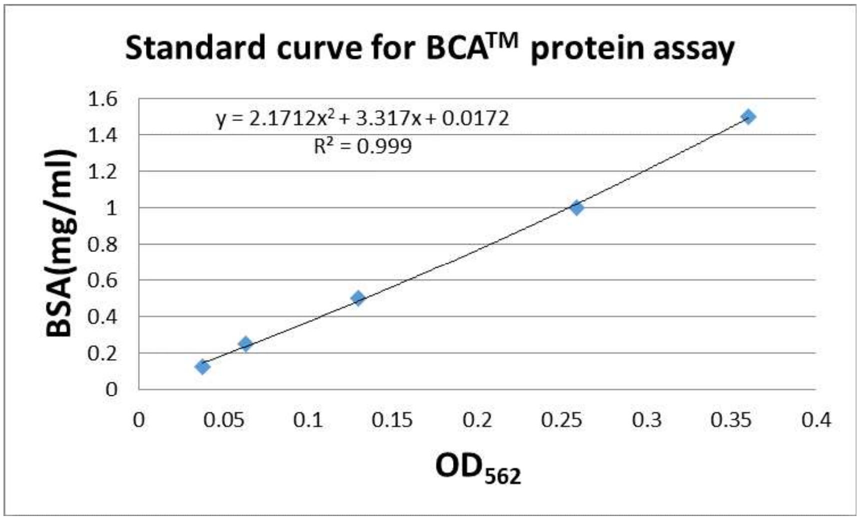

Sample	OD_{562} (diluted 4 times)	Concentration
PROT4 O_H	0.217	3.35 mg/ml

For research use only

SDS-PAGE & Western blot Analysis: Chimera 2

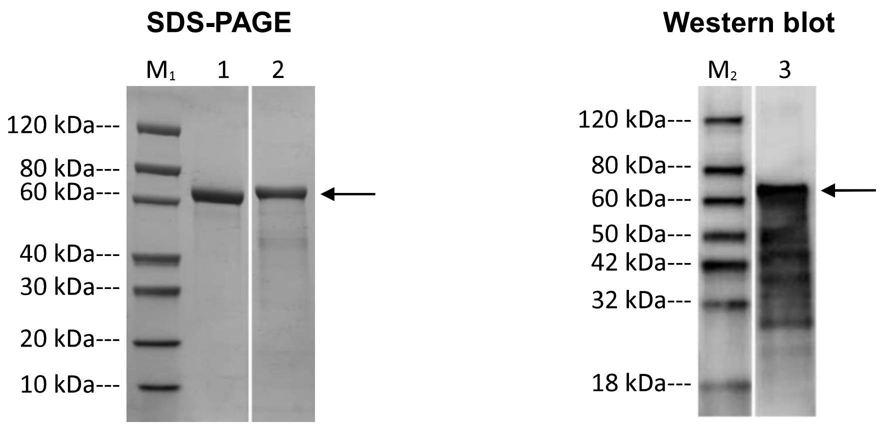

Fig.1 SDS-PAGE and Western blot analysis of PROT1
Lane M_1: Protein Marker, GenScript, Cat. No. M00516
Lane M_2: Protein Marker, GenScript, Cat. No. M00521
Lane 1: BSA (2.00 μg)
Lane 2: PROT1 (Reducing condition, 2.00 μg)
Lane 3: PROT1 (Reducing condition)
Primary antibody: Mouse-anti-His mAb (GenScript, Cat.No. A00186)

Quantification--BSA Standard Curve:

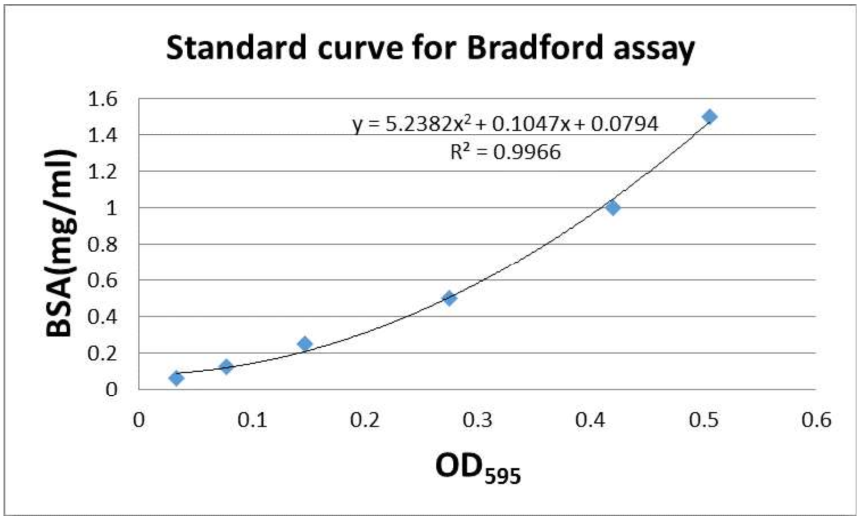

Sample	OD_{595} (diluted 3 times)	Concentration
PROT1	0.360	2.38 mg/ml

For research use only
